# Accessibility and Decision-Supportive Quality of Benign Breast Disease Articles on Chinese Hospital WeChat Public Accounts: Nationwide Cross-Sectional Study

**DOI:** 10.2196/95994

**Published:** 2026-10-09

**Authors:** Jing Li, Chenxi He, Shi Wang, Lichi Ke, Hongfan Yu, Xing Wei, Cheng Lei, Dehua Hu, Zhiliang Zhang, Wen Zhou, Min Li, Wenlin Wu, Wei Xu, Jin Bai, Qiuling Shi

**Affiliations:** 1 State Key Laboratory of Ultrasound in Medicine and Engineering, Chongqing Medical University, No. 1, Medical College Road, Yuzhong District, Chongqing, 400016, China, 86 18290585397; 2 School of Clinical Medicine, Chongqing Medical and Pharmaceutical College, Chongqing, China; 3 School of Public Health, Chongqing Medical University, Chongqing, China; 4 Department of Breast and Thyroid Surgery, People's Hospital of Jinniu District, Chengdu, China; 5 Department of Pharmacy, the First Affiliated Hospital of Army Medical University (Third Military Medical University), Chongqing, China; 6 Department of Thoracic Surgery, Sichuan Clinical Research Center for Cancer, Sichuan Cancer Hospital & Institute, Sichuan Cancer Center, University of Electronic Science and Technology of China, Chengdu, China; 7 Lueyang County Maternal and Child Health and Family Planning Service Center, Lueyang County, Shanxi, China; 8 Breast Center, Three Gorges Hospital of Chongqing University, Chongqing, China

**Keywords:** telemedicine, benign breast disease, social media, shared decision-making, patient participation, WeChat

## Abstract

**Background:**

Shared decision-making (SDM) is critical for managing clinically ambiguous conditions such as benign breast disease (BBD), requiring reliable health information. In China, WeChat public accounts (WPAs) run by hospitals serve as a major health information channel, yet their quality remains unevaluated.

**Objective:**

This study aimed to evaluate the quality of BBD-related articles on hospital WPAs in China.

**Methods:**

Using a multistage stratified random sampling approach, we conducted a nationwide survey examining the quality of articles published from January 2020 to November 2024. The articles were evaluated for accessibility (readability, image quality, and user-friendliness) and SDM readiness (credibility, balance, and decision relevance) using validated instruments and content analysis.

**Results:**

Among 276,617 articles from 158 sampled hospitals, 464 were related to BBD. Accessibility was generally good: 72.4% (336/464) were written at a junior high school reading level. Visual presentation was acceptable, although only 10.2% (42/410) had complete image labeling. The Patient Education Materials Assessment Tool for Printable Materials (PEMAT-P) scores showed moderate understandability (mean 73.6%, SD 17.3%) but limited actionability (mean 50%, SD 23.2%). For SDM readiness, the mean Quality Evaluation Scoring Tool (QUEST) score was 14.9 (SD 4.7) out of 28, with treatment-related articles scoring lower than prevention-related articles (*P*<.001). In the balance evaluation, using Brief DISCERN, 84.3% (182/216) of articles were below the high-quality threshold, often lacking information on treatment risks and quality-of-life impacts. Decision relevance revealed variability in surgical recommendations, especially regarding excision criteria and pregnancy-related guidance.

**Conclusions:**

WPA articles on BBD showed good accessibility but substantial gaps in SDM support, particularly in information balance and decision relevance. Standardized guidelines and quality improvements are needed to enhance patient-centered online health services.

## Introduction

Benign breast disease (BBD) poses a major clinical challenge, accounting for 55% to 90% of all breast lesions [1-3]. The risk of subsequent breast cancer varies considerably, depending on the degree of epithelial proliferation and atypia [4-6]. Unlike malignancy, BBD lacks evidence-based management protocols and often requires individualized strategies such as surveillance, biopsy, surgery, or follow-up [7,8]. This clinical uncertainty necessitates preference-sensitive care, where comparable treatment options require decisions tailored to patient-specific risks and informed preferences [9-11]. Shared decision-making (SDM), recommended by the American Society of Breast Surgeons and European guidelines [7,12], is particularly relevant for younger female patients, who constitute most BBD cases [13-15]. Evidence shows that SDM in surgical care reduces decisional conflict, anxiety, and unnecessary procedures while improving the overall quality of care and reducing costs [16-19]. Despite these well-documented benefits, the routine adoption of SDM in clinical practice remains suboptimal [20]. A critical barrier worldwide is the difficulty of integrating patient decision aids into clinical workflows, particularly in resource-constrained settings [21,22].

Telemedicine plays a supportive and interactive role in facilitating decision-making [23]. In China, the national “Internet + Health Care” initiative has rapidly expanded the model of internet hospitals, with 3340 internet hospitals nationwide as of September 2024 [24]. WeChat public accounts (WPAs), China’s dominant social media platform with 1.385 billion active users, have been adopted by most tertiary hospitals to integrate professional expertise with broad digital reach [25,26]. Notably, more than 60% of Chinese health consumers prefer WeChat as their primary source of medical information, highlighting the potential of WPAs as tools for patient education and decision support [27-29]. Among the vast and varied health information available online, hospital-operated WPAs carry unique weight as official institutional voices and tend to be regarded by patients as more credible channels for medical guidance. As time-constrained clinical consultations increasingly drive patients to seek health information online, the BBD-related content patients encounter on WPAs before visits may shape the knowledge, expectations, and preferences they bring into clinical encounters, with potential implications for the starting point and quality of SDM [30,31].

Despite this promise, concerns persist regarding the quality and relevance of WPA content for decision-making [25,32]. Although prior research has evaluated breast cancer information on public platforms such as Twitter and YouTube, a systematic assessment of BBD-related content on hospital-based social media is lacking [33,34]. To address this gap, our nationwide study evaluates whether hospital-based WPAs provide accessible, decision-supportive content that enables informed patient participation in SDM, offering novel insights into digital health communication within China’s emerging internet hospital ecosystem.

## Methods

### Study Design and Sampling

A nationwide, multistage random sampling approach was used across 7 geographic regions in China. In each region, 3 provinces were randomly selected, and 2 municipal-level cities were randomly chosen per province (Table S1 in Multimedia Appendix 1). Following the Hospital Classification Management Measures [35], 4 hospitals were sampled per city: 1 maternal and child health (MCH) hospital, 1 traditional Chinese medicine (TCM) hospital, and 2 general hospitals (including 1 tertiary and 1 secondary).

Articles published between January 1, 2020, and November 30, 2024, were collected via automated web scraping with manual verification. Chinese keyword searches identified BBD-related content (Table S2 in Multimedia Appendix 1). Articles explicitly addressing BBD were included, while those addressing malignancy only, administrative or promotional content, or unrelated topics were excluded (Table S3 in Multimedia Appendix 1).

### Data Extraction and Screening

Data were extracted at both institutional and article levels. Briefly, institutional data—including hospital characteristics, geographic location, bed capacity, and digital engagement—were sourced from official websites and the National Health Commission database. Article-level data—including publication date, view count, and article content—were obtained directly from the sampled WeChat (Tencent Holdings) public accounts. All article view counts were captured on March 16, 2025; views per day were calculated as cumulative views divided by the number of calendar days since publication.

### Evaluation Framework and Assessment Tools

Following established criteria for evaluating online health information [36] and the International Patient Decision Aid Standards (IPDAS) [37], we developed a structured framework encompassing 2 major domains: accessibility and readiness (Figure 1).

**Figure 1.**
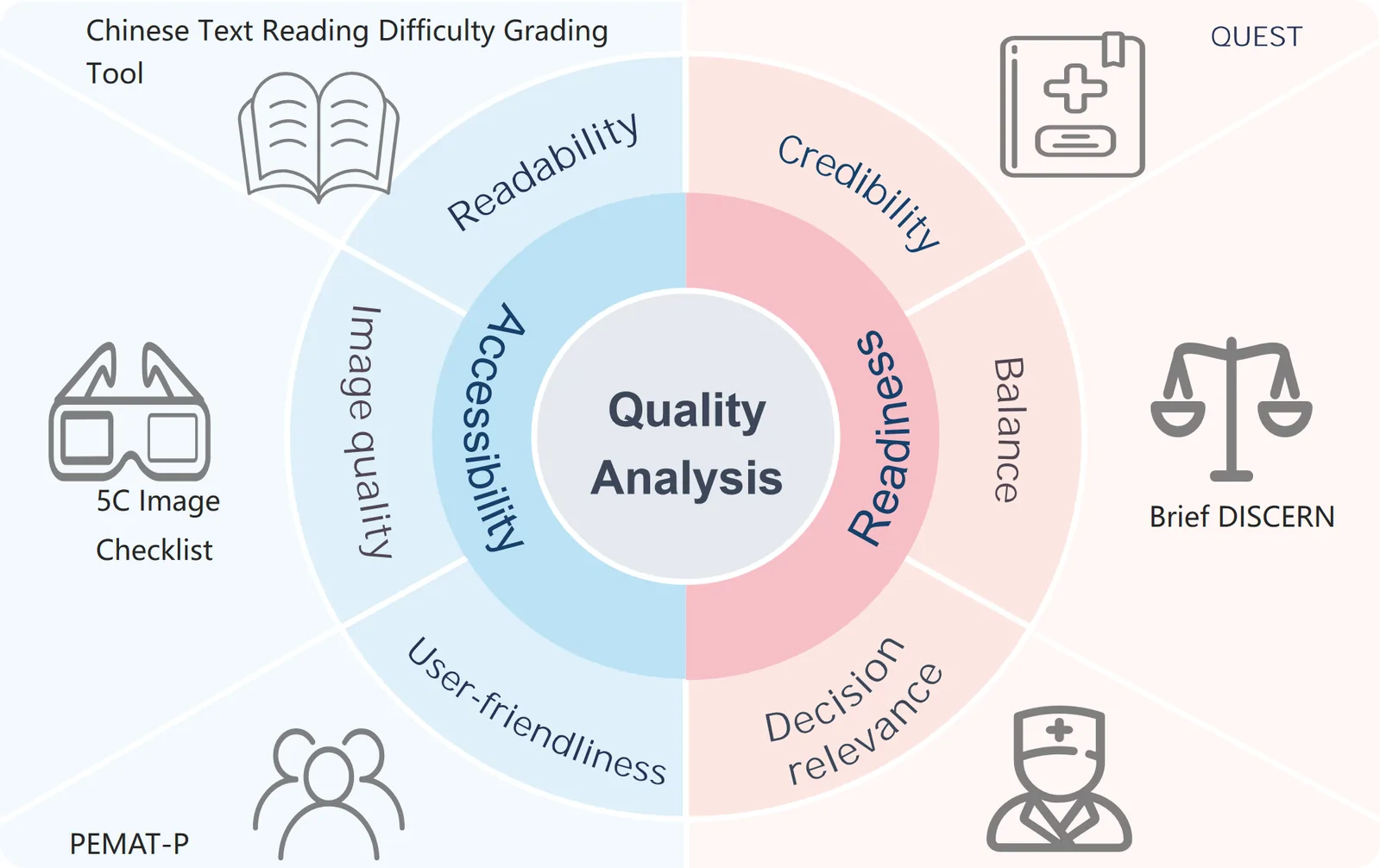
Conceptual framework for evaluating benign breast disease articles on Chinese hospital WeChat public accounts (2020‐2024). PEMAT-P: Patient Education Materials Assessment Tool for Printable Materials; QUEST: Quality Evaluation Scoring Tool.

#### Accessibility

Accessibility assessed how clearly and effectively health information was delivered and the extent to which the materials supported patient engagement. It comprised 3 dimensions: readability, image quality, and user-friendliness.

First, readability was assessed using the Chinese Text Reading Difficulty Grading Tool, which applies natural language processing techniques to estimate text difficulty based on linguistic features from the Chinese textbook corpus [38,39]. Readability scores were automatically computed for each article without requiring human raters.

Second, image quality was assessed using the 5C Image Checklist, adapted from the Centers for Disease Control and Prevention’s (CDC) Simply Put guide [40,41]. The checklist evaluates 5 domains: clarity, contribution, contradiction, caption, and cultural appropriateness. Each article was independently rated by 2 trained reviewers (SW and CH), with discrepancies resolved through discussion.

Finally, user-friendliness was evaluated with the Chinese version of the Patient Education Materials Assessment Tool for Printable Materials (PEMAT-P), including understandability and actionability, with scores reported as continuous percentages (higher values indicate better quality) [42]. To incorporate patient-centered perspectives, 20% (43/216) of treatment-related articles were randomly selected and independently evaluated by a panel of 14 adult patients using the PEMAT-P tool. Evaluators were recruited by convenience sampling from hospitals and had no prior experience in evaluating patient education materials. Their ages ranged from 23 to 42 years; all held bachelor’s or master’s degrees, and 6 had a personal history of benign breast nodules. Each article was assessed by 2 independent evaluators, with each evaluator reviewing approximately 6 articles.

#### Readiness

Readiness reflected the extent to which article content could support patients’ participation in SDM. It was assessed across 3 dimensions: credibility, balance, and decision relevance.

First, credibility was measured using the Quality Evaluation Scoring Tool (QUEST) [43], covering 6 domains (authorship, attribution, conflict of interest, currency, complementarity, and tone; score range 0-28). Assessments were performed by 2 doctoral students in biomedical engineering with clinical training (JL and WZ).

Second, balance was evaluated using the Brief DISCERN, a validated short version of the DISCERN tool designed for treatment information [44,45]. Six items were assessed: information source, information currency, treatment mechanism, treatment benefits, treatment risks, and impact on quality of life. A score of ≥16 indicated high quality [45]. Two surgeons (LK and ZZ) independently assessed all treatment-related articles.

Finally, decision relevance was examined through directed content analysis of (1) clinical contexts for which treatment was advised and (2) treatment modalities suggested [46]. A coding guide was iteratively developed from 10% (22/216) of treatment-related articles and refined through consensus discussion between 2 reviewers (JL and XW). The final coding guide is presented in Table S4 in Multimedia Appendix 1. All 216 treatment-related articles were then independently double-coded, and discrepancies were resolved through discussion.

### Statistical Analysis

Descriptive statistics summarized article and hospital characteristics. Categorical variables were presented as frequencies and percentages. Continuous variables were reported as means (SDs) for normally distributed data or medians (IQR) for skewed data. Chi-square tests were used for categorical variables. Skewed continuous variables were compared using the Kruskal-Wallis test and Bonferroni-corrected Dunn post hoc tests. Interrater reliability for all continuous quality scores (QUEST, Brief DISCERN, and PEMAT-P) was assessed using 2-way random-effects intraclass correlation coefficients for single-rating, absolute agreement (ICC_2,1_). For the categorical variables (5C Image Checklist), Cohen kappa was calculated for each domain. For decision relevance content analysis, percentage agreement was used (with an ≥85% threshold).

Linear regression was used to assess temporal trends in publication volume, with year as the independent variable. Given the nested structure of the data (articles clustered within hospitals), linear mixed-effects models with hospital as a random intercept were fitted using the *lme4* package in R (version 4.5.0; R Foundation for Statistical Computing). *P* values were obtained by likelihood ratio tests. A 2-sided *P* value <.05 was considered statistically significant. NVivo (version 20; QSR International) was used for qualitative data analysis. Datawrapper (Datawrapper GmbH) was used to visualize geographic distribution. Origin 2025 (OriginLab) was used for data visualization.

### Ethical Considerations

This study was approved by the ethics committee of Chongqing University Three Gorges Hospital (KLS2025122). As all WPA articles assessed were publicly available and contained no identifying information, the ethics committee granted a waiver of informed consent. Adult patients participated in article evaluation with oral consent. All data were anonymized, and no compensation was provided.

## Results

### Study Sample and Article Selection

Multistage random sampling yielded 40 city-level units after excluding 2 units: Turpan (Xinjiang) had no independent TCM hospital, and Haibei Prefecture (Qinghai) had no tertiary general hospital at the time of sampling. Of the 160 hospitals expected under the sampling protocol, 158 were included (Figure 2). From these hospitals, 265 WPAs were identified (Figure 2), from which 276,617 articles published between January 2020 and November 2024 were retrieved. After keyword screening, 5526 potentially relevant articles were identified, and 464 BBD-related articles met the inclusion criteria.

**Figure 2.**
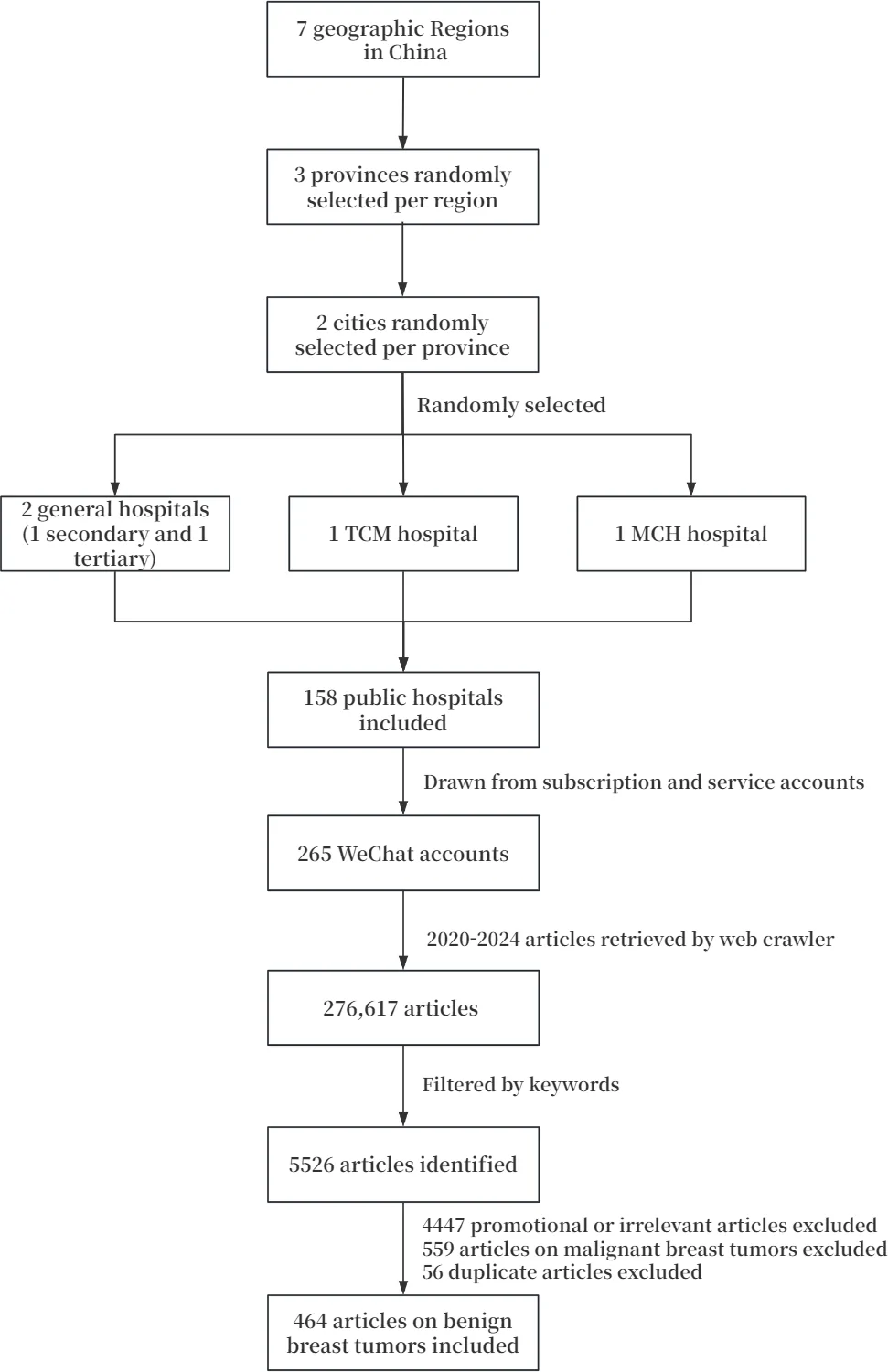
Flowchart of article screening for the nationwide cross-sectional survey of benign breast disease content on Chinese hospital WeChat public accounts. MCH: maternal and child health; TCM: traditional Chinese medicine.

### Hospital Characteristics and Digital Engagement

The sample comprised 39 tertiary general hospitals, 40 secondary general hospitals, 39 TCM hospitals, and 40 MCH hospitals (Table 1). Overall, 94 (59.5%) hospitals were tertiary-level, and 64 (40.5%) were secondary-level.

In terms of digital engagement, 155 (98.1%) hospitals operated WPAs, achieving full coverage among tertiary general and TCM hospitals. In contrast, only 65.2% (103/158) maintained official websites, ranging from 89.7% (35/39) of tertiary hospitals to 27.5% (11/40) of secondary general hospitals. Certification for internet hospitals was limited (40/158, 25.3%), with the highest proportion among tertiary general hospitals (22/39, 56.4%).

Over the past 5 years, publication volumes on WPAs showed no significant regional differences across China (*P*>.05; Figure S1 in Multimedia Appendix 1). By hospital type, the median 5-year publication volume ranged from 271 (IQR 83-759) in secondary general hospitals to 743 (IQR 188-1250) in MCH hospitals; secondary general hospitals had significantly lower publication volumes than the other 3 hospital types (Table 1).

**Table 1.** Baseline characteristics of Chinese public hospitals sampled across 7 geographic regions (N=158).

Characteristics	Total hospitals	Tertiary general hospitals (n=39)	Secondary general hospitals (n=40)	Traditional Chinese medicine hospitals (n=39)	Maternal and child health hospitals (n=40)
Hospital profile					
Tertiary hospital, n (%)	94 (59.5)	39 (100)	0 (0)	30 (76.9)	25 (62.5)
Beds available, mean (SD)	1058 (980)	2132 (1015)	388 (214)^a^	1046 (751)	589 (563)^b^
Digital engagement, n (%)					
Internet hospital	40 (25.3)	22 (56.4)	2 (5)	10 (25.6)	6 (15)
Hospitals with official websites	103 (65.2)	35 (89.7)	11 (27.5)	31 (79.5)	27 (67.5)
Hospitals with WeChat accounts	155 (98.1)	39 (100)	38 (95)	39 (100)	39 (97.5)
Annual publication volume per hospital, median (IQR)^c^	562 (156-1213)	724 (95-1345)	271 (83-759)	680 (300-1586)	743 (188-1250)

a Beds available in secondary general hospitals were significantly lower than those in both tertiary general hospitals and traditional Chinese medicine hospitals (Kruskal-Wallis test followed by Dunn post hoc tests with Bonferroni corrections; *P*<.05).

b Beds available in maternal and child health hospitals were significantly lower than those in both tertiary general hospitals and traditional Chinese medicine hospitals (Kruskal-Wallis test followed by Dunn post hoc tests with Bonferroni corrections; *P*<.05).

c Publication volumes differed significantly among hospital categories (Kruskal-Wallis H=15.520; *P*=.001). Of the 6 pairwise Dunn post hoc comparisons with Bonferroni correction, 3 remained statistically significant: secondary general hospitals had significantly lower publication volumes than tertiary general hospitals (adjusted *P*=.01), traditional Chinese medicine hospitals (adjusted *P*<.001), and maternal and child health hospitals (adjusted *P*=.03).

### Quality Assessment of BBD-Related Articles

#### Overview

Of the 158 hospitals in the overall sample, 103 contributed at least 1 eligible BBD-related article, yielding 464 articles for analysis. Among these, 248 (53.4%) focused on prevention and 216 (46.6%) on treatment. Linear regression confirmed a significant upward trend in annual publication volume over the 5-year study period (*β*=15.7 articles per year, 95% CI 6.0-25.4; *P*=.01; Figure S2 in Multimedia Appendix 1).

#### Readability

The mean reading grade level was 12.9 (SD 1.7, range 6-20) years. Among these materials, 15.7% (73/464) were written at a primary school level, the majority (336/464, 72.4%) at a junior high school level, and 11.9% (55/464) at a senior high school level, indicating that the overall text complexity was generally acceptable and appropriate for the literacy level of the general population (Table 2).

**Table 2.** Multidimensional quality assessment of benign breast disease articles on Chinese hospital WeChat public accounts.^a^

Dimensions and attributes	Articles
Readability (n=464)	
Reading grade level (years), mean (SD; range)	12.9 (1.7; 6-20)
Reading grade level, n (%)	
Primary school level (age group: 6-11 years)	73 (15.7)
Junior high school level (age group: 12-14 years)	336 (72.4)
Senior high school level (age group: 15-18 years)	55 (11.9)
Image quality (n=410), n (%)	
Images clearly recognizable	399 (97.3)
Image reinforcing the text	348 (84.9)
Image consistent with the text	408 (99.5)
Images with captions	42 (10.2)
Image culturally appropriate	408 (99.5)
User-friendliness (%; Patient Education Materials Assessment Tool for Printable Materials; n=43), mean (SD)	
Understandability	73.6 (17.3)
Actionability	50.0 (23.2)
Credibility (n=464)	
QUEST^b^ score, mean (SD)	14.9 (4.7)
QUEST score groups, n (%)	
≤7	34 (7.3)
8-14	133 (28.7)
15-21	277 (59.7)
22-28	20 (4.3)
Balance (n=216)	
Brief DISCERN score, median (IQR)	12.5 (10.5-14.5)
Brief DISCERN score groups, n (%)	
6-10	53 (24.5)
11-15	129 (59.7)
16-20	34 (15.7)
21-30	0 (0)

a Readability and Quality Evaluation Scoring Tool (QUEST) scores were calculated based on all included articles (n=464); image quality assessment was based on articles containing images (n=410); Brief DISCERN scores were based on treatment-related articles (n=216); and Patient Education Materials Assessment Tool for Printable Materials (PEMAT-P) scores were based on a randomly selected subsample of 43 treatment-related articles.

b QUEST: Quality Evaluation Scoring Tool.

#### Image Quality

Of 410 articles containing images, most visuals were clear (n=399, 97.3%) and consistent with the text (n=408, 99.5%), and 84.9% (n=348) reinforced the content. However, image labeling was inadequate: only 10.2% (n=42) included fully labeled captions, 23.7% (n=97) included partially labeled captions, and 66.1% (n=271) had no labels. Nearly all images (n=408, 99.5%) were culturally appropriate. Cohen κ for the 5C Image Checklist ranged from 0.847 to 1.000 across the 5 domains.

#### User-Friendliness

Forty-three articles, randomly sampled from 216 (20%) treatment-related articles, were included. The interrater intraclass correlation coefficient (ICC_2,1_) was 0.51. Mean understandability was 73.6% (SD 17.3%), and mean actionability was 50% (SD 23.2%).

#### Credibility

The mean QUEST score was 14.9 (SD 4.7). Only 4.3% (20/464) of articles scored >21, while 36% (167/464) of articles scored ≤14 (Table 2). Interrater agreement for the QUEST total score was 0.79 (ICC_2,1_). Prevention-related articles scored higher than treatment-related articles (mean 15.8, SD 3.8 vs mean 13.8, SD 5.3; *P*<.001; Table S5 in Multimedia Appendix 1).

In multivariable analysis, article content was the only factor independently associated with credibility (*β*=−2.16; *P*<.001). Neither hospital level nor service mode showed a significant independent association with QUEST scores. For article dissemination, all 3 factors were independently associated with views per day: tertiary hospitals (*β*=0.38; *P*=.02), internet hospitals (*β*=0.35; *P*=.008), and treatment-related articles (*β*=0.20; *P*=.006) each showed higher readership (Table S5 in Multimedia Appendix 1).

#### Balance

The median Brief DISCERN score for treatment-related articles (n=216) was 12.5 (IQR 10.5-14.5), with 84.3% (182/216) falling below the high-quality threshold of 16 (Table 2). Interrater reliability for the total Brief DISCERN score was 0.57 (ICC_2,1_). Source information was rarely adequate (17/216, 7.8% scoring ≥3), and only 3.2% (7/216) of articles achieved scores ≥3 for currency. Mechanisms and benefits were addressed in more than 50% of articles (scoring ≥2), whereas risks and quality-of-life impacts were addressed in only 12.5% (27/216, scoring ≥2; Figure 3).

**Figure 3.**
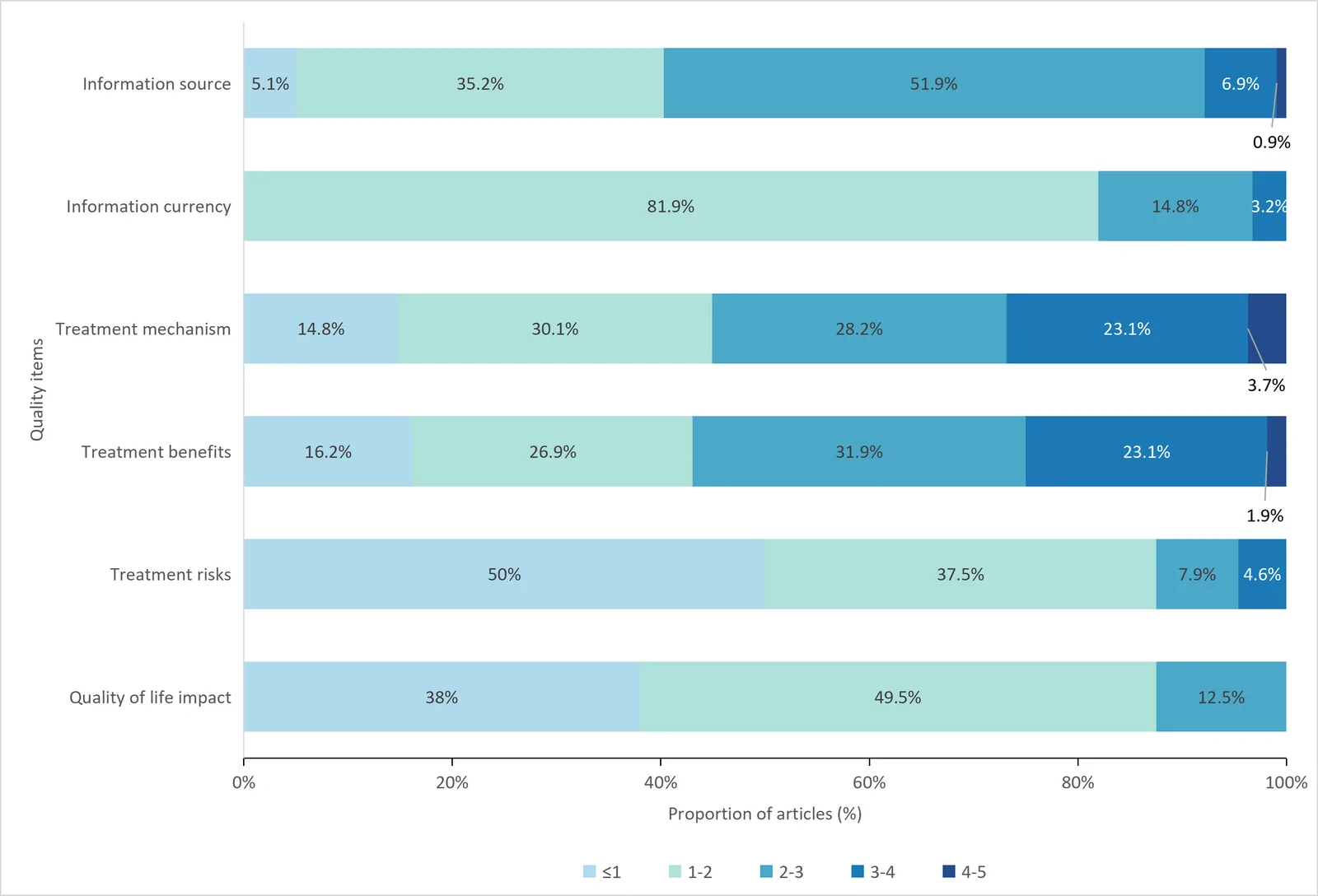
Distribution of Brief DISCERN scores across 6 items among 216 treatment-related benign breast disease articles on Chinese hospital WeChat public accounts.

#### Decision Relevance

Treatment-related articles (n=216) most often described surgery, particularly vacuum-assisted excision (VAE; 57/216, 26.4%) and open surgical excision (OSE; n=45, 20.8%; Figure 4A). Minimally invasive or ablative techniques (eg, focused ultrasound, microwave, and radiofrequency) were rarely mentioned. TCM therapies appeared in 17.6% (38/216) of articles, most often oral medications (n=17, 7.9%) and acupuncture (n=10, 4.6%; Figure 4B). Strengths were consistently emphasized for VAE, while limitations were noted mainly for OSE and seldom for VAE or focused ultrasound (Figure S3 in Multimedia Appendix 1). The coding reproducibility between the 2 independent reviewers across the entire dataset was 90%.

**Figure 4.**
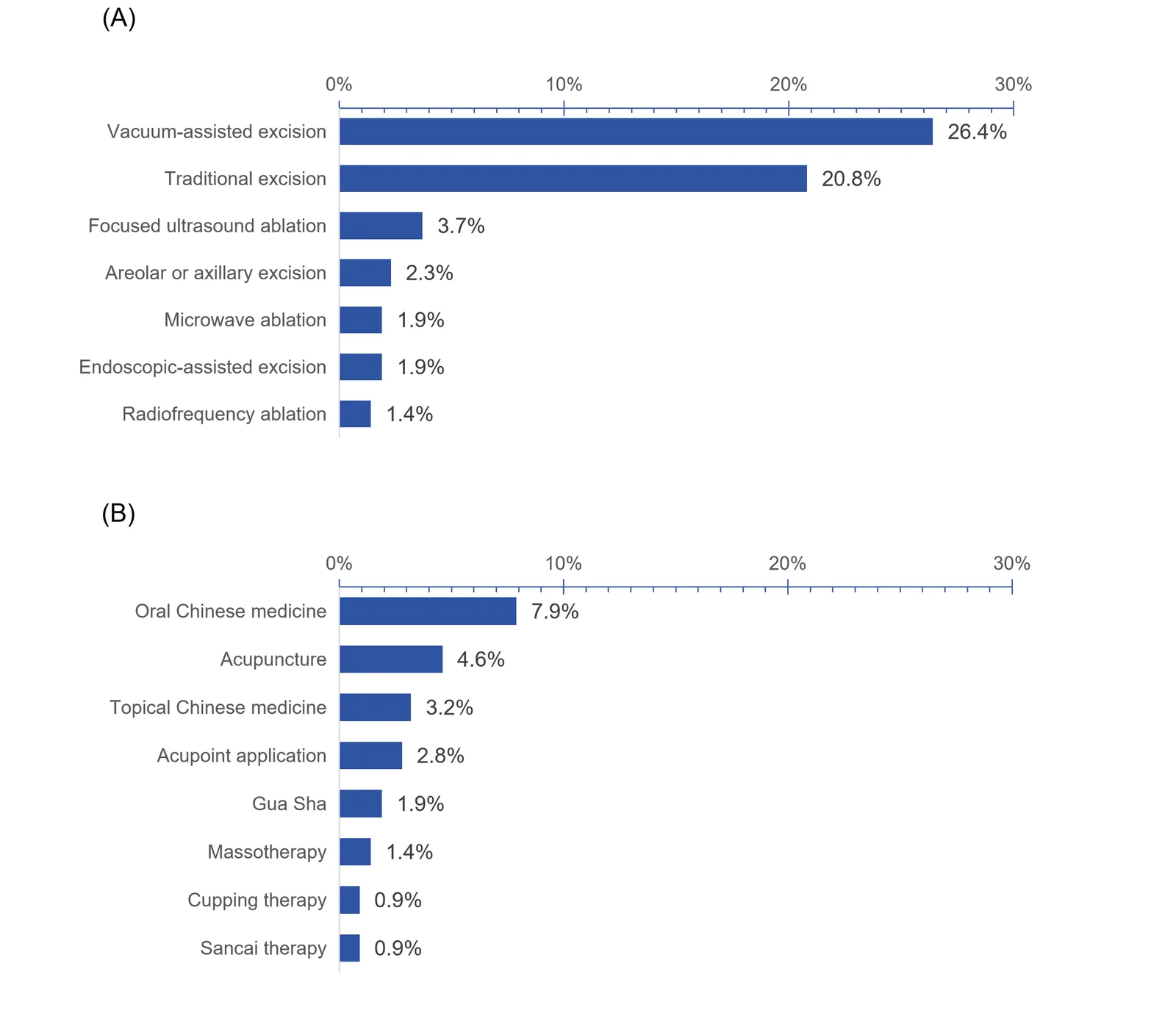
Distribution of treatment approaches for benign breast diseases in 216 treatment-related WeChat articles from Chinese public hospitals. (A) Frequency of surgical treatment approaches and (B) frequency of traditional Chinese medicine–based treatment approaches. Percentages indicate the proportion of articles that mentioned each treatment method.

Clinical indications for surgery were inconsistently reported. Tumor-related factors were most frequently mentioned (n=76, 35.2%), including growth, pathology, size, suspicion of malignancy, and imaging features. Patient-related factors were noted in 25 (11.6%) articles, including pregnancy planning, family history, and age, as well as concerns such as anxiety or cosmetic issues. Detailed distributions are shown in Figure 5.

**Figure 5.**
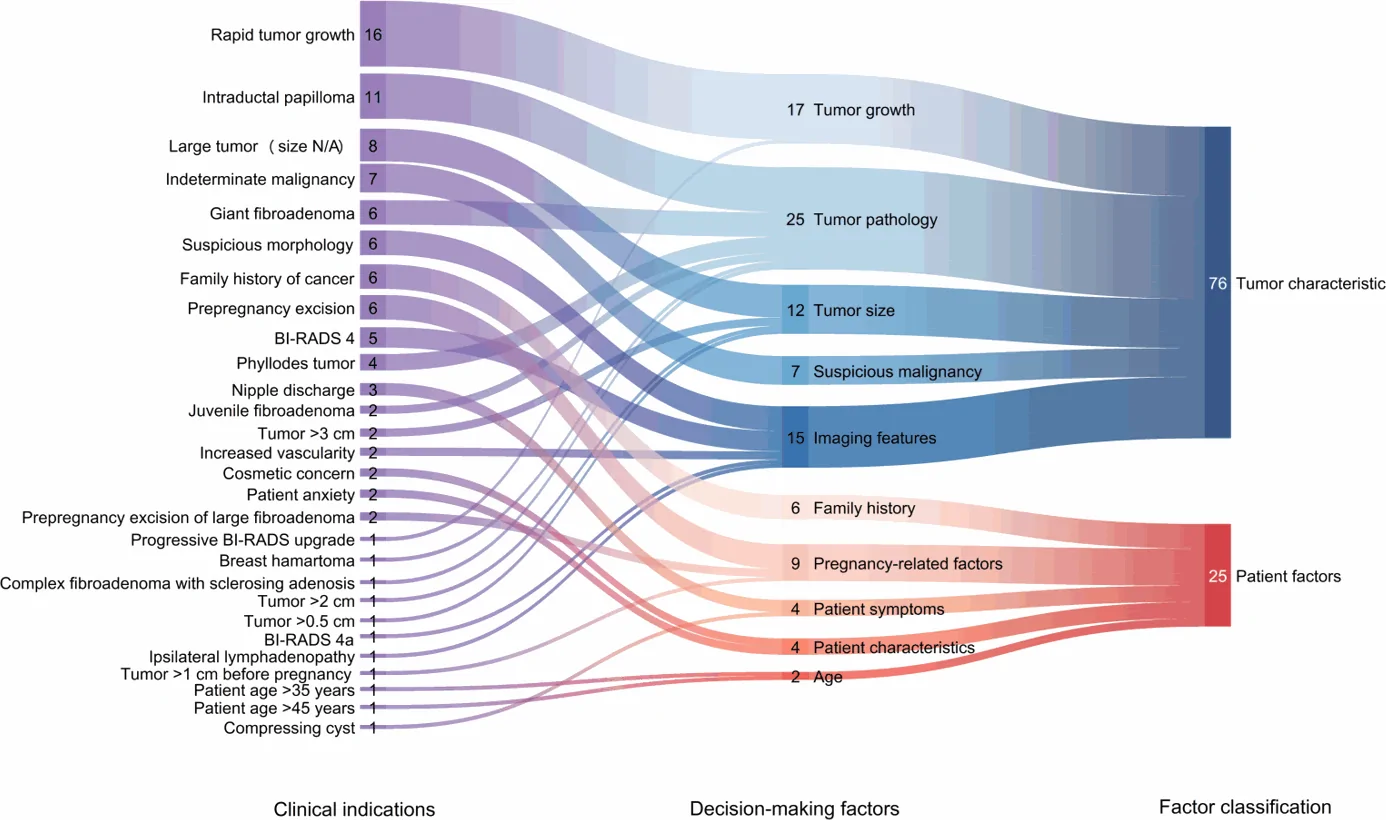
Directed content analysis of surgical recommendations for benign breast nodules in 216 treatment-related WeChat articles from Chinese public hospitals. BI-RADS: Breast Imaging Reporting and Data System; N/A: not available.

## Discussion

### Principal Findings

Using a nationwide random sampling approach, we evaluated the accessibility and SDM readiness of hospital WPAs in the context of BBD. This study, the first to explore this issue in China, revealed that WPA articles were generally accessible and had the potential to extend SDM beyond clinical encounters. However, the risk-benefit balance was often insufficient, and decision relevance was limited.

### Availability for Health Information Dissemination

SDM in China is still in its early stage, and successful implementation requires culturally appropriate pathways that are customized to fit the local clinical and resource context [47]. Telemedicine can facilitate SDM, and the rapid development of internet hospitals in China provides a new avenue by allowing patients to access information before consultations [48]. In our study, nearly all sampled hospitals operated WPAs, and approximately one-fourth were certified for internet health care, highlighting their commitment to digital service delivery. As part of the expanding digital health ecosystem, public hospitals are increasingly using online platforms such as WPAs to disseminate medical information and engage with patients [49].

Although decision aids are implemented across diverse areas of health care, multiple studies have consistently highlighted significant barriers to their routine use in clinical practice, including time burden and lack of incentives [50]. Effective decision support should convey the right information to the right person in the right format through the right channel and at the appropriate point in the workflow [51]. Incorporating the patient portal into SDM delivery is a promising strategy, as it does not rely on clinicians' initiation [52-54]. This asynchronous SDM also extends decision-making through written or pictorial exchanges across space and time [55,56]. Developing WeChat-based integration tools could therefore represent a pragmatic strategy for embedding SDM into clinical practice.

### Accessibility for Patient Engagement

Despite digital health care platforms providing significant opportunities for SDM, information accessibility remains a critical factor in their effective use. Overall, WPA articles demonstrated acceptable accessibility. Most materials were written at a junior high school level, which was appropriate for the general population, although patients with low health literacy may still face challenges [57]. Visual aids contribute to enhancing patients’ comprehension of health information [58-60]. In our study, visual aids were generally suitable, and optimizing captions and explanatory labels would further strengthen their educational value. From a user-friendliness perspective, materials were generally understandable, but actionability was limited. Although patients could follow the presented information, there was little explicit guidance on what steps to take or how to engage in the decision-making process [40].

These findings align with evidence that hospital WPAs function as hybrid platforms integrating health service delivery with health information dissemination; however, their role has yet to fully extend from providing information to actively supporting patient decision-making [61]. In addition, incorporating multimedia formats—such as images, videos, and interactive tools—can improve accessibility, clarify patient preferences, and foster more effective participation in decision-making [62-64]. Emerging digital technologies, including AI-enabled recommendation systems, may further support personalized decision-making and adherence [65,66].

### Readiness for SDM

Effective SDM requires not only the availability of health information but also high-quality and SDM-supportive content [67]. In surgical decision-making for benign tumors, it is crucial for physicians and patients to engage in SDM, with patients receiving balanced information to help them make fully informed choices regarding treatment options [13].

However, balance in treatment-related articles on BBD emerged as a major concern, with only 15.7% (34/216) of articles meeting the criteria for high-quality content. Most articles lacked balanced discussions of potential risks or quality-of-life impacts, both of which are essential for informed decision-making [34]. This aligns with findings from US hospital websites, showing that such imbalanced information could mislead patients and undermine the SDM process [68]. Ensuring that the content patients encounter before consultations is balanced and decision-relevant could make WPAs a practical vehicle for embedding SDM into routine telemedicine.

Patients often rely on clinicians for information, yet inconsistencies arising from institutional practices or individual physician experience may lead to patient confusion [13,69]. Our content analysis revealed wide variation in treatment recommendations, which was primarily reflected in 2 aspects: treatment approaches and surgical decision-making. Most articles presented surgical indications as a combination of tumor characteristics (size and growth rate) and patient-specific factors (symptoms, age, family history, and anxiety), generally supported by literature [4,70-72]. However, the specific thresholds for tumor size that necessitate surgical excision and the definition of “rapidly growing” varied considerably. For instance, the recommended size threshold for fibroadenoma ranged from 2 cm to 3 cm across different regional and international guidelines [70,73,74], resulting in inconsistent information in the WPA articles.

Considerations related to pregnancy were also presented inconsistently. Due to the visible changes in the mammary glands in response to hormonal stimuli, breast masses detected during pregnancy require timely evaluation and individualized management [75,76]. Some of the included WPA articles suggested that surgical management of BBD in the context of pregnancy tends to be proactive in China, which may be partly related to the accessibility of specialized medical services and the anxiety of these patients [77-79]. Women with breast issues often fear the possibility of breast cancer, resulting in unnecessary treatments, patient anxiety, and expenditures [3,80].

Treatment approaches discussed in WeChat articles exhibit a hybrid model that integrates Western medicine and TCM in China [81]. Although VAE remains the dominant surgical approach, emerging treatments such as focused ultrasound also show promising potential [82]. Although TCM has demonstrated benefits in symptom relief and postoperative recovery, its clinical efficacy in treating BBD requires further validation through high-quality studies [83,84]. In the context of BBD, where management options range from observation to intervention, it is important for patients to receive balanced information regarding the risks and benefits of these approaches. Delivering patient-centered care can enhance patient satisfaction and adherence and reduce overtreatment and its adverse impact on quality of life [64,85,86].

### Strengths and Limitations

#### Strengths

This study has several strengths. First, it adopted a nationwide sample covering diverse hospital types and levels of public hospitals across China. Second, it is the first systematic evaluation of the quality and SDM-supportive potential of BBD-related health education articles on hospital WPAs. Finally, a comprehensive assessment framework combining validated tools was applied, incorporating both clinician and patient perspectives to strengthen the reliability of the findings.

#### Limitations

First, the analysis was restricted to publicly available WPA articles and official institutional data, which may not fully reflect clinical communication and may contain reporting delays. Clinician and patient perspectives could complement these findings in future work. Second, retrospective web scraping may have missed deleted articles, although survivorship bias is likely limited given the large sample and the rarity of content removal by hospital WPAs. Third, the study assessed information quality rather than clinical accuracy. BBD management recommendations vary across guidelines, and future accuracy audits may be informative where consensus exists. Finally, interrater reliability for PEMAT-P was moderate, attributable to the diverse backgrounds of the 14 patient evaluators and the inherent subjectivity of judging understandability, which varies with individual health literacy and experience. The user-friendliness estimates thus warrant cautious interpretation, and more robust assessment tools and approaches are needed to complement the current evaluation.

### Conclusions

Hospital WPAs have the potential to support SDM for BBD, owing to the availability and accessibility of medical information. However, their current SDM readiness is limited, underscoring the need for standardization and quality improvement initiatives to optimize patient-centered decision-making.

## Supplementary material

10.2196/95994 Multimedia Appendix 1 Supplementary tables and figures for the benign breast disease article analysis.

## Abbreviations

BBD: benign breast disease
CDC: Centers for Disease Control and Prevention
ICC_2,1_: 2-way random-effects intraclass correlation coefficients for single-rating, absolute agreement
IPDAS: International Patient Decision Aid Standards
MCH: maternal and child health
OSE: open surgical excision
PEMAT-P: Patient Education Materials Assessment Tool for Printable Materials
QUEST: Quality Evaluation Scoring Tool
SDM: shared decision-making
TCM: traditional Chinese medicine
VAE: vacuum-assisted excision
WPA: WeChat public account
