# Supplementary material for: Accessibility and Decision-Supportive Quality of Benign Breast Disease Articles on Chinese Hospital WeChat Public Accounts: Nationwide Cross-Sectional Study
Source: JMIR Med Inform. 2026 Oct 9;14:e95994. doi: 10.2196/95994 (PMC13652390; doi:10.2196/95994)
Supplement: Supplementary file 1 [file medinform-v14-e95994-s001.docx]

**Supplementary Materials**

**Table of Contents**

Supplementary Table 1. List of sampled provinces by geographic-economic region

Supplementary Table 2. Search terms and results for benign breast disease articles

Supplementary Table 3. Classification criteria for article screening

Supplementary Table 4. Codebook for Directed Content Analysis of Treatment-Related Information in Benign Breast Disease Articles

Supplementary Table 5. Article characteristics and multivariable linear mixed-effects models (N = 464 articles, 103 hospitals)

Supplementary Figure 1. Average publication volume over a 5-year period by hospital WeChat accounts across different regions in China

Supplementary Figure 2. Annual publication volume of benign breast disease articles

Supplementary Figure 3. Reporting of strengths and limitations of surgical methods in benign breast disease articles

**Supplementary Table 1. List of sampled provinces by geographic-economic region**

| Geographic regions | Provinces included (sampled provinces in bold) |
| --- | --- |
| East China | Shandong, Jiangsu, Anhui, Zhejiang, Fujian, Shanghai, Jiangxi |
| South China | Guangdong, Guangxi, Hainan |
| Central China | Hubei, Hunan, Henan |
| North China | Beijing, Tianjin, Hebei, Shanxi, Inner Mongolia |
| Northwest China | Ningxia, Xinjiang, Qinghai, Shaanxi, Gansu |
| Southwest China | Sichuan, Yunnan, Guizhou, Tibet, Chongqing |
| Northeast China | Liaoning, Jilin, Heilongjiang |

Notes: Three provinces were randomly selected from each region, and two cities were randomly chosen from each selected province, resulting in a total of 21 provinces and 40 cities (Beijing and Chongqing are municipalities directly under the Central Government, and no further prefecture-level sampling was conducted).
**Supplementary Table 2. Search terms and results for benign breast disease articles**

| Search Strategy | Search Scope | Search Content (Chinese) | Search Results (n) |
| --- | --- | --- | --- |
| Exact phrase search | All content | 乳腺结节, 乳腺包块, 乳腺肿块, 乳腺肿物, 乳腺良性肿瘤, 乳腺纤维腺瘤, 乳腺纤维瘤, 乳腺占位, 乳房结节, 乳房包块, 乳房肿块, 乳房肿物, 乳房良性肿瘤, 乳房纤维腺瘤, 乳房纤维瘤, 乳房占位 | 1728 |
| Combined keyword search | Content | Disease-related: 乳腺&结节, 乳腺&肿物, 乳腺&肿块, 乳腺&纤维瘤, 乳腺&纤维腺瘤, 乳腺&包块 | 5669 |
|  |  | Disease-related: 乳房&结节, 乳房&肿物, 乳房&肿块, 乳房&纤维瘤, 乳房&纤维腺瘤, 乳房&包块 | 2602 |
|  |  | Treatment-related: 乳腺&聚焦超声, 乳腺&海扶, 乳腺&HIFU, 乳腺&旋切, 乳腺&消融 | 1597 |
|  |  | Treatment-related: 乳房&聚焦超声, 乳房&海扶, 乳房&HIFU, 乳房&旋切, 乳房&消融 | 554 |
|  | Title | Treatment-related: 安珂, 麦默通, 旋切, HIFU, 海扶, 聚焦超声 | 114 |
|  |  | Disease-related: 乳腺结节, 乳腺包块, 乳腺肿块, 乳腺肿物, 乳腺良性肿瘤, 乳腺纤维腺瘤, 乳腺纤维瘤, 乳腺占位, 乳房结节, 乳房包块, 乳房肿块, 乳房肿物, 乳房良性肿瘤, 乳房纤维腺瘤, 乳房纤维瘤, 乳房占位 | 109 |
| Total articles retrieved | | | 12373 |
| Final unique articles after removing duplicates | | | 5526 |

Notes:

· **Chinese terms represent all possible expressions for breast nodules/benign breast diseases.**

· “**&” indicates combined keyword search within article content.**

**Exact phrase searches included terms as continuous strings; combined keyword searches allowed discontinuous appearance of terms.**

**Supplementary Table 3. Classification criteria for article screening**

| Eligibility | Category | Definition | Notes |
| --- | --- | --- | --- |
| Inclusion | Benign breast tumors | Articles addressing benign or borderline breast tumors. | Includes **fibroadenoma**, **intraductal papilloma**, **phyllodes tumor**, **hamartoma**, **breast cysts**, **lipoma**, and **mammary gland hyperplasia**. Articles mentioning both benign and malignant tumors were also included. |
|  | **– Prevention** | Articles focusing on prevention or early detection strategies. | Including symptoms, causes, screening of benign lesions. |
|  | **– Treatment-related** | Articles focusing on treatment or management of benign tumors. | Including medication, surgery, traditional Chinese medicine, observation, and diagnostic procedures such as biopsy. |
| **Exclusion** | Malignant breast tumors | Articles exclusively addressing malignant breast tumors (e.g., Paget’s disease). | Articles that do not reference benign lesions and solely address breast cancer were excluded. |
|  | Promotional content | Articles primarily for publicity or institutional announcements. | Including news releases, physician profiles, or event notices without substantive medical content. |
|  | Irrelevant or non-breast conditions | Articles on conditions unrelated to breast tumors or focusing on other systems. | Examples include Mondor’s disease, nipple inversion, precocious puberty, neonatal breast enlargement, breast tuberculosis, gynecomastia, accessory breast, macromastia, mastitis, and non-breast nodules (e.g., thyroid, lung, liver). |

**Supplementary Table 4. Codebook for directed content analysis of treatment-related information in benign breast disease articles**

**Coding Manual Overview**

| No. | Level | Node / Code Definition | Inclusion Criteria | Exclusion Criteria | Coding Example |
| --- | --- | --- | --- | --- | --- |
| A | 1 | 治疗方式 (Treatment Modalities) Articles discussing treatment strategies, principles, or modalities for benign breast disease (BBD). | Any paragraph that discusses how BBD should be treated, including specific modalities, general strategies, or comparative treatment approaches. | Content solely about disease definition, diagnosis, or prevention without reference to treatment. |  |
| A1 | 2 | 西医治疗方式 (Western Medicine Treatment Modalities) Conventional interventions for BBD, including surgery and minimally invasive procedures. | Any mention of a specific Western medical treatment for BBD, including procedural names, or technique descriptions. | TCM modalities (coded under A2); general health advice without a named treatment. |  |
| A1.1 | 3 | 乳腺微创旋切手术 (Vacuum-Assisted Excision, VAE / Minimally Invasive Rotational Excision) A minimally invasive biopsy-excision procedure using vacuum-assisted devices (e.g., Mammotome, EnCor) to remove benign breast lesions under ultrasound guidance through a small incision. | Mentions of VAE, Mammotome, EnCor, vacuum-assisted biopsy/excision, or 微创旋切 for breast masses; descriptions of procedure, indications, or outcomes. | Core needle biopsy without vacuum assistance. |  |
| A1.1.1 | 4 | 优点 (Advantages of VAE) Reported benefits or strengths of vacuum-assisted excision. | Text describing: small incision, quick recovery, minimal scarring, good cosmetic outcome, outpatient procedure, high accuracy, simultaneous diagnosis and treatment. | Disadvantages (coded under A1.1.2). | e.g., Mammotome minimally invasive surgery leaves only a 3mm incision without obvious scarring; patients can be discharged the same day. |
| A1.1.2 | 4 | 缺点 (Disadvantages of VAE) Reported drawbacks or limitations of VAE. | Text describing: higher cost, risk of hematoma, risk of residual lesion, requires specialized equipment, not suitable for lesions near skin or chest wall. | Advantages (coded under A1.1.1). | e.g., VAE has limited applicability for superficial lesions close to the skin. |
| A1.2 | 3 | 乳腺肿块切除手术或传统开放手术 (Open Surgical Excision / Traditional Open Surgery) Conventional open surgical removal of breast masses via a skin incision directly over the lesion. | Mentions of open surgery, traditional excision, lumpectomy for benign lesions, descriptions of procedure or indications. | Core needle biopsy; mastectomy for malignancy; cosmetic breast surgery without mass excision. |  |
| A1.2.1 | 4 | 优点 (Advantages of Open Surgery) Reported benefits or strengths of open surgical excision. | Text describing: complete lesion removal, wide applicability, lower equipment requirements, suitable for large or superficial masses, definitive pathology, lower recurrence rate. | Disadvantages (coded under A1.2.2). | e.g., Traditional open surgery achieves complete mass removal and is particularly suitable for large or superficially located lesions. |
| A1.2.2 | 4 | 缺点 (Disadvantages of Open Surgery) Reported drawbacks or limitations of open surgery. | Text describing: larger scar, longer recovery, more postoperative pain, visible cosmetic deformity, requires hospitalization in some cases. | Advantages (coded under A1.2.1). | e.g., Open surgery leaves a longer incision and may affect postoperative breast appearance. |
| A1.3 | 3 | 聚焦超声消融手术 (Focused Ultrasound Surgery, FUS / HIFU) A non-invasive thermal ablation technique using high-intensity focused ultrasound to destroy targeted breast lesions without skin incision. | Mentions of HIFU, focused ultrasound for breast masses; descriptions of procedure or principles. | Diagnostic ultrasound; physical therapy ultrasound; HIFU for non-breast conditions. |  |
| A1.3.1 | 4 | 优点 (Advantages of FUS) Reported benefits or strengths of FUS. | Text describing: non-invasive, no incision, no scar, outpatient procedure, preserves breast appearance, real-time imaging guidance, quick recovery. | Disadvantages (coded under A1.3.2). | e.g., FUS requires no surgical incision; extracorporeal focused ultrasound ablates the lesion while preserving breast appearance. |
| A1.3.2 | 4 | 缺点 (Disadvantages of FUS) Reported drawbacks or limitations of FUS. | Text describing: high equipment cost, limited availability, not suitable for all lesion types, requires MRI guidance in some protocols. | Advantages (coded under A1.3.1). | e.g., FUS equipment is expensive, and this technique is currently available in only a few large hospitals. |
| A1.4 | 3 | 环乳晕或腋下切口乳腺肿瘤切除术 (Periareolar or Transaxillary Incision Breast Tumor Resection) Surgical excision of breast masses through a circumareolar or axillary incision to minimize visible scarring on the breast surface. | Mentions of periareolar incision, axillary incision, for breast mass removal; references to cosmetic or hidden-scar approaches. | Standard open surgery without specification of incision location; endoscopic surgery (coded under A1.5). | e.g., Using a periareolar incision, the mass is removed along the areolar border where the scar remains inconspicuous. |
| A1.5 | 3 | 乳腺腔镜手术 (Endoscopic Breast Surgery) Minimally invasive breast surgery using an endoscope through small remote incisions (e.g., axillary approach), allowing magnified visualization and precise dissection. | Mentions of endoscopic breast surgery, mastoscopic surgery; descriptions of endoscopic technique for breast lesion removal. | Open surgery; VAE/vacuum-assisted biopsy; endoscopic surgery for non-breast conditions. | e.g., Endoscopic breast surgery accesses the lesion through a small axillary incision, providing magnified visualization for precise dissection. |
| A1.6 | 3 | 微波消融术 (Microwave Ablation, MWA) A thermal ablation technique using microwave energy delivered via a needle electrode to destroy breast lesions through coagulative necrosis. | Mentions of microwave ablation, 微波消融 for breast tumors; technical descriptions, indications, or outcomes. | MWA for non-breast organs (e.g., liver, lung, thyroid) without breast-specific context. | e.g., Microwave ablation delivers microwave energy through a needle electrode into the tumor, inducing coagulative necrosis. |
| A1.7 | 3 | 射频消融术 (Radiofrequency Ablation, RFA) A thermal ablation technique using radiofrequency current delivered via an electrode needle to destroy breast lesions through thermal necrosis. | Mentions of radiofrequency ablation, 射频消融 for breast tumors; technical descriptions, indications, or outcomes. | RFA for non-breast organs without breast context; microwave ablation (coded under A1.6). | e.g., RFA uses high-frequency electrical current to generate heat, causing coagulative necrosis of tumor tissue. |
| A2 | 2 | 中医治疗方式 (Traditional Chinese Medicine Treatment Modalities) TCM-based interventions for BBD, including herbal medicine, acupuncture, external applications, and manual therapies. | Any mention of a specific TCM treatment modality for BBD; names of TCM formulas, acupoints, or techniques targeting breast conditions. | General wellness or dietary advice without a named TCM method. |  |
| A2.1 | 3 | 中药口服（中成药）(Oral Chinese Herbal Medicine / Proprietary Chinese Medicine) Internal administration of Chinese herbal decoctions, granules, or proprietary Chinese medicines for treating BBD. | Mentions of specific Chinese herbal formulas, patent medicines (e.g., Rupixiao, Xiaoyao Wan, Xiaojin Wan), or herbal decoctions for breast masses/hyperplasia. | General references to TCM without specifying oral herbal treatment; dietary therapy without herbal formula. | e.g., Xiaoyao San has the effects of soothing the liver, relieving depression, resolving phlegm, and dispersing masses; it is commonly used for mammary gland hyperplasia. |
| A2.2 | 3 | 针灸治疗 (Acupuncture and Moxibustion) Treatment of BBD using needle acupuncture, electroacupuncture, moxibustion, or related acupoint stimulation techniques. | Mentions of acupuncture, electroacupuncture, moxibustion, acupoint stimulation for breast conditions; specific acupoints used (e.g., Danzhong CV17, Rugen ST18, Qimen LR14). | General meridian theory without actual treatment description. | e.g., Needling Danzhong (CV17), Rugen (ST18), and Qimen (LR14) can regulate the Chong and Ren meridians and disperse masses. |
| A2.3 | 3 | 中药外敷 (External Herbal Application) Application of Chinese herbal pastes, ointments, or compresses directly to the breast to treat BBD. | Mentions of herbal paste, ointment, compress for breast masses; specific herbal compositions for external use. | Oral herbal medicine; acupoint patching (coded under A2.4). | e.g., A paste made from frankincense, myrrh, and rhubarb is applied externally to promote blood circulation and disperse swelling. |
| A2.4 | 3 | 穴位贴敷 (Acupoint Herbal Patching) Application of herbal patches to specific acupoints for treating BBD, combining acupoint stimulation with transdermal herbal delivery. | Mentions of acupoint patching, 穴位贴敷, acupoint application for breast conditions; specific acupoints and herbal formulas used. | General external herbal application without acupoint targeting; acupuncture. | e.g., Herbal patches applied to Danzhong and Rugen acupoints exert dual effects through acupoint stimulation and transdermal drug delivery. |
| A2.5 | 3 | 刮痧 (Gua Sha / Scraping Therapy) A TCM manual technique using a scraping tool on lubricated skin to stimulate circulation and relieve stagnation for treating BBD. | Mentions of gua sha, specifically for breast or chest conditions; descriptions of technique or target areas (e.g., back regions corresponding to breasts). | Gua sha for general wellness. | e.g., Gua sha on the corresponding back region of the breasts can unblock meridians, resolve stasis, and disperse masses. |
| A2.6 | 3 | 推拿按摩 (Tuina / Therapeutic Massage) TCM manual manipulation techniques applied to the breast, chest, or related meridian pathways to treat BBD. | Mentions of tuina, massage, specifically for breast masses. | General relaxation massage without therapeutic intent for breast conditions. | e.g., Local breast tuina combined with acupoint pressing along the Liver and Stomach meridians can regulate qi movement, disperse masses, and relieve pain. |
| A2.7 | 3 | 拔罐 (Cupping Therapy) Application of suction cups to the skin, typically on the back along the Bladder meridian, to promote qi and blood circulation for treating BBD. | Mentions of cupping for breast conditions; description of target areas on the back or chest. | Cupping for non-breast conditions; cupping without therapeutic context for BBD. | e.g., Sliding cupping along the Bladder meridian on the back can unblock meridians and regulate organ function to assist treatment of mammary gland hyperplasia. |
| A2.8 | 3 | 三才疗法 (Sancai Therapy) A comprehensive TCM approach integrating three modalities (typically oral herbal medicine, external application, and device-based physiotherapy) for breast disease management. | Mentions of 三才疗法, Sancai therapy for breast conditions; description of the combined modality approach. | Single TCM modality without reference to the Sancai combination framework. | e.g., Sancai therapy combines oral herbal medicine, acupoint patching, and instrument-based physiotherapy to treat benign breast disease through multiple pathways. |
| B | 1 | 建议手术的情况 (Situations Recommending Surgery) Clinical contexts, criteria, or risk factors discussed as indications or justifications for surgical intervention in BBD. | Any paragraph discussing reasons, criteria, or circumstances under which surgery is recommended or should be considered for BBD. | General treatment descriptions without specifying surgical indication; discussions of surgery being unnecessary or contraindicated. |  |
| B1 | 2 | BI-RADS 4类 (BI-RADS Category 4) Recommendation for excision based on BI-RADS category 4 findings (suspicious abnormality). | Text stating that BI-RADS 4 lesions require biopsy or surgical excision; mentions of BI-RADS 4 as a surgical indication. | BI-RADS 3 or lower without surgical recommendation; BI-RADS mentioned only for diagnostic classification without treatment implication. | e.g., BI-RADS category 4 lesions carry a 2%-95% risk of malignancy; surgical or core needle biopsy is recommended for definitive diagnosis. |
| B2 | 2 | BI-RADS 4a类 (BI-RADS Category 4a) Recommendation for surgical intervention based on BI-RADS 4a findings (low suspicion for malignancy). | Text specifically mentioning BI-RADS 4a as an indication for surgery or biopsy decision-making; discussion of the threshold for intervention. | BI-RADS 4b or 4c without specific 4a discussion. | e.g., Although BI-RADS 4a lesions have a low probability of malignancy (2%-10%), biopsy or excision is still recommended. |
| B3 | 2 | 具体肿瘤 (Specific Tumor Types) Surgical recommendation based on specific tumor types with malignant potential (e.g., phyllodes tumors, atypical hyperplasia, intraductal papilloma). | Text recommending surgery for phyllodes tumor, intraductal papilloma, fibroadenoma, hamartoma, breast cyst, etc. | - | e.g., Phyllodes tumors, intraductal papillomas, large fibroadenomas, and symptomatic breast cysts are generally recommended for surgical excision. |
| B4 | 2 | 不良影像因素 (Adverse Imaging Features) Surgical recommendation based on suspicious imaging characteristics such as irregular margins, spiculation, microcalcifications, or interval growth. | Text citing specific imaging features (irregular shape, spiculated margins, microcalcifications, increased vascularity, posterior acoustic shadowing) as grounds for surgical excision. | - | e.g., Breast nodules with irregular shape and spiculated margins on ultrasound should raise suspicion of malignancy; surgical excision is recommended. |
| B5 | 2 | 分级参考因素 (Factors Considered in BI-RADS Grading) Factors that inform BI-RADS classification and thereby influence surgical decision-making (e.g., lesion size, patient age, menopausal status). | Text describing that a higher BI-RADS category indicates greater malignancy risk and therefore serves as a criterion for considering surgical intervention. | Direct enumeration of BI-RADS classification features without treatment implications. | e.g., The higher the BI-RADS category, the greater the malignancy risk; category 4 and above generally require surgery for definitive diagnosis. |
| B6 | 2 | 生长迅速的标准 (Criteria for Rapid Growth) Definition and clinical thresholds for growth velocity that prompt surgical intervention. | Text defining rapid growth (e.g., volume increase >20% in 6 months, diameter increase >20% per year); statements that rapidly growing lesions require excision. | - | e.g., Breast nodules showing a volume increase greater than 20% within 6 months should be considered for surgical excision. |
| B7 | 2 | 建议手术其他因素 (Other Factors Recommending Surgery) Additional clinical or patient-related factors prompting surgical recommendation (e.g., patient anxiety, pain, pregnancy planning, cosmetic concerns, family history of breast cancer). | Text citing patient anxiety, persistent pain, planned pregnancy, strong family history of breast cancer, cosmetic concerns, or patient preference as reasons for surgery. | - | e.g., For women planning pregnancy who have breast nodules, pre-pregnancy surgical excision is recommended to avoid hormone-stimulated growth during gestation. |

**Supplementary Table 5. Article characteristics and multivariable linear mixed-effects models (N = 464 articles, 103 hospitals)**

A. Descriptive characteristics and univariate comparisons

| Category | Subcategory | Number (%) | QUEST score, Mean±SD | p | Article Views/day, M (IQR) | p |
| --- | --- | --- | --- | --- | --- | --- |
| Hospital level | Tertiary hospitals | 407 (87.7%) | 15.1±4.5 | 0.022 | 1.82 (0.63–4.62) | 0.001 |
|  | Secondary hospitals | 57 (12.3%) | 13.1±5.3 |  | 0.41 (0.14–1.14) |  |
| Service mode | Internet hospitals | 208 (44.8%) | 15.6±4.4 | 0.167 | 2.51 (0.87–7.62) | <0.001 |
|  | Non-internet hospitals | 256 (55.2%) | 14.3±4.8 |  | 0.98 (0.30–2.44) |  |
| Article content | Prevention-related | 248 (53.4%) | 15.8±3.8 | <0.001 | 1.11 (0.39–3.29) | 0.008 |
|  | Treatment-related | 216 (46.6%) | 13.8±5.3 |  | 1.90 (0.64–5.12) |  |

B. Multivariable linear mixed-effects models

| Predictor | QUEST β (SE) | 95% CI | QUEST p | log(Views/day)β (SE) | 95% CI | log(Views/day) p |
| --- | --- | --- | --- | --- | --- | --- |
| Hospital level (tertiary vs secondary) | 1.31 (0.77) | [-0.20, 2.83  ] | 0.088 | 0.38 (0.16) | [0.07, 0.69  ] | 0.015 |
| Service mode (internet vs non-internet) | 0.57 (0.61) | [-0.65, 1.77  ] | 0.35 | 0.35 (0.13) | [0.09, 0.61  ] | 0.008 |
| Article content (treatment vs prevention) | −2.16 (0.41) | [-2.96, -1.36  ] | <0.001 | 0.20 (0.07) | [0.06, 0.34  ] | 0.006 |

*Notes:* Panel A *P* values from univariate linear mixed-effects models with hospital-level random intercepts. Panel B *P* values from a single multivariable model with all predictors entered simultaneously, reflecting independent effects after mutual adjustment. All models estimated by maximum likelihood.

**Supplementary Figure 1. Average publication volume over a 5-year period by hospital WeChat accounts across different regions in China.**

|  | East China | South China | Central China | North China | Northwest China | Southwest China | Northeast China |
| --- | --- | --- | --- | --- | --- | --- | --- |
| Average publications per account (mean±SD) | 963±862 | 886±1884 | 866±1008 | 1259±1616 | 1314±1997 | 853±667 | 992±1555 |

Notes:* No statistically significant differences in average publication volumes among the seven regions (p>0.05).

**Supplementary Figure 2. Annual publication volume of benign breast disease Articles**

Number of Articles

Year-on-Year Growth (%)

|  | Number | YoY Growth (%) |
| --- | --- | --- |
| 2020 | 66 |  |
| 2021 | 73 | 10.6061% |
| 2022 | 84 | 15.0685% |
| 2023 | 120 | 42.8571% |
| 2024/11 | 121 | 0.833333% |

**Supplementary Figure 3. Reporting of strengths and limitations of surgical methods in Benign Breast Disease articles.**

Number of Articles

| Surgical Methods Mentioned in Articles (n = 216) | Strength reported | Limitation reported |
| --- | --- | --- |
| VAE 57 | 52 | 4 |
| OSE 45 | 7 | 29 |
| FUS 8 | 8 | 1 |

VAE: vacuum-assisted excision; OSE: open surgical excision; FUS: focused ultrasound. Strengths refer to the reported advantages or benefits of each surgical method, while limitations refer to the reported risks, disadvantages, or shortcomings
